# Survey and Identification of Fusarium Head Blight Pathogens of Wheat in the Western Cape Region of South Africa

**DOI:** 10.3390/pathogens14010080

**Published:** 2025-01-16

**Authors:** Ali Al-Hashimi, Augustine Innalegwu Daniel, Omolola Aina, Morné Du Plessis, Marshall Keyster, Ashwil Klein

**Affiliations:** 1 Plant Omics Laboratory, Department of Biotechnology, Faculty of Natural Sciences, University of the Western Cape, Robert Sobukwe Road, Bellville 7535, South Africa; 4178288@myuwc.ac.za (A.A.-H.); 4112896@myuwc.ac.za (A.I.D.); 4177137@myuwc.ac.za (O.A.); 2 Department of Biochemistry, School of Life Sciences, Federal University of Technology, P.M.B 65, Minna 920101, Niger State, Nigeria; 3 Genetics Department, University of the Free State, Bloemfontein 9301, South Africa; duplessismg@ufs.ac.za; 4 Environmental Biotechnology Laboratory, Department of Biotechnology, Faculty of Natural Sciences, University of the Western Cape, Robert Sobukwe Road, Bellville 7535, South Africa; mkeyster@uwc.ac.za

**Keywords:** disease severity, food security, *Fusarium* head blight, mycotoxin, *Fusarium graminearum* species complex, Western Cape

## Abstract

*Fusarium* head blight (FHB) is a major disease affecting wheat production worldwide, caused by multiple *Fusarium* species. In this study, seven *Fusarium* strains were isolated from wheat fields across the Western Cape region of South Africa and identified through phylogenetic analysis. The strains were classified into three species complexes: the *Fusarium graminearum* species complex (FGSC), *Fusarium incarnatum-equiseti* species complex (FIESC), and *Fusarium tricinctum* species complex (FTSC). Disease severity was highest in the South coast regions of Swellendam (42.73%) and Caledon (38.00%), with the dough stage of wheat development showing the highest disease rate (0.3 in Swellendam and Caledon). The phylogenetic analysis showed distinct clustering of these isolates with known species from the NCBI database, confirming their classification. *F. ipomoeae* was uniquely found in Swellendam and Caledon, while *F. tricinctum* occurred only in Klipheuwel and Caledon, highlighting geographical variation in species distribution. Mycotoxin profiling revealed that *F. culmorum* and *F. pseudograminearum* produced zearalenone, *F. culmorum* and *F. tricinctum* produced 15-acetyl-deoxynivalenol (15-ADON), while *F. pseudograminearum* produced nivalenol (NIV). These findings provide significant insights into the distribution of *Fusarium* species and their associated trichothecene chemotypes in the Western Cape, which is crucial for developing effective FHB management strategies and ensuring food security and safety.

## 1. Introduction

Fusarium head blight (FHB), also known as head scab, is a major and devastating fungal disease of wheat (*Triticum aestivum*) globally [1]. The disease is caused by the *Fusarium graminearum* species complex (FGSC), the fourth-most scientifically and economically significant fungal phytopathogen worldwide [2]. The disease affects farm output, such as yield and grain quality, resulting in a significant loss of grain due to mycotoxin contamination, which can affect human and animal health [3]. Annually, approximately USD 3 billion is invested annually for the control and management of this disease in the USA, and about 7 million hectares of land has been reported to be severely affected by FHB epidemics in China [1,2]. In South Africa, FHB has been a cause of concern for many years, causing a lot of economic loss both to the farmers and the government [2]. In the late 80s, around KwaZulu-Natal, there was a reported case of a disease incidence of over 70% [2]. Since then, there have been efforts both by the government and individual farmers to control this disease. Nevertheless, these efforts have yet to achieve the desired result because resistant wheat varieties, bio-fungicides, and licensed fungicides are not available [3]. Nonetheless, the genomics age has brought about a great deal of advancement, especially in the areas of identifying resistance genes and quantitative trait loci (QTLs), highlighting resistance mechanisms and molecular breeding for enhanced resistance [4]. Despite these achievement and advancement made over the years, overcoming FHB disease still poses serious challenges since resistance to the *Fusarium* species is complex [5].

*Fusarium* species associated with FHB produce mycotoxins, and the most common mycotoxins detected in wheat samples from FHB-infected fields were deoxynivalenol (DON), deoxynivalenol-3-glucoside (D3G), enniatin B (ENN B), enniatin B1 (ENN B1), culmorin, 15-hydroxyculmorin, and aurofusarin [6]. *Fusarium graminearum*, associated with FHB in small grain crops, has been reported to produce deoxynivalenol (DON) [7]. In a study on winter wheat in Slovakia, *Fusarium graminearum*, *F. avenaceum*, *F. culmorum*, *F. ipomoea,* and *Microdochium nivale* were isolated from the ears of wheat infected with FHB, with *F. graminearum* being the most frequently isolated species [8].

Globally, there are no holistic approaches or measures towards controlling FHB disease. Most studies only aim at various aspects of the disease through identifying the causative agents. For example, studies have shown that various mycotoxins/metabolites associated with FHB such as deoxynivalenol (DON), zearalenone (ZEN), and enniatin (ENN) were identified out of 216 wheat samples that were analyzed [6]. In another study carried out by Powell et al. [9], *Sphaerodes mycoparasitica,* a specific biocontrol, considerably decreased the occurrence of DON in harvested wheat grains [9]. Qiu et al. [10] described the accurate identification of FHB-infected wheat kernels using Raman spectroscopy and deep learning networks, offering a quick and non-invasive diagnostic technique [10]. Additionally, another study by Matić et al. [11] to investigate the effects of *Fusarium* inoculation and nitrogen fertilization on the defense response in wheat spikes stressed the potential of breeding wheat varieties with enhanced defense mechanisms against FHB. Lastly, Spanic et al. [12] studied the protein components and antioxidant enzymes implicated in the FHB infection process. This study shows that resistant wheat varieties had lower DON accumulation and higher peroxidase activity.

Rain with high temperature during anthesis and an abundance of primary inoculum are risk factors for the occurrence of FHB in wheat, which affects wheat yield in South Africa [2]. Significant precipitation, lower temperature at night, and increased humidity during flowering are important contributors to elevated infection levels [13]. FHB epidemics, which can result in yield losses of up to 80%, are facilitated by these conditions [14]. When seasonal environmental conditions are favorable, there is a significant increase in the rate of the disease, particularly in regions where conservation tillage is practiced and maize is produced in a double cropping system with wheat [15]. Farmers find it difficult to control the disease because flowering is characterized by unpredictable weather [16,17].

There are several factors that affect FHB in wheat. Various *Fusarium* species, including *F. graminearum*, *F. culmorum*, and *F. avenaceum*, which have different mycotoxin profiles and geographic adaptations, are the sources of the disease [13,18]. In South Africa, several *Fusarium* species are responsible for FHB in wheat. For instance, 24 species from seven broad species complexes, including *F. sambucinum complexes* (FSAMSCs), *F. incarnatum-equiseti species complexes* (FIESCs), *F. chlamydosporum* (FCSC), *F. fujikuroi* (FFSC), *F. oxysporum* (FOSC), *F. solani* (FSSC), and *F. tricinctum* species complexes (FTSCs), were identified during a detailed characterization of FHB pathogens in 2008 and 2009 [1]. FGSC, which accounted for 75.7% of the isolates, was the predominant *Fusarium* species in each of the four irrigation regions [1]. With 16 *Fusarium* species from all seven species complexes, the Northern Cape has the highest species diversity [19]. Moreover, *F. brachygibbosum*, *F. asiaticum*, *F. lunulosporum*, and *F. transvaalense* were identified worldwide, and *F. acuminatum*, *F. armeniacum*, *F. avenaceum*, *F. temperatum*, and *F. pseudograminearum* were identified for the first time in South Africa [2,20,21].

Fusarium head blight (FHB) epidemics are influenced by several factors, including weather conditions such as stormy days with warm temperatures during anthesis, which promote the abundance of primary inoculum [22]. The type of organic mulch used can also affect the presence of *Fusarium* species in the soil prior to wheat flowering and the mycotoxin content in mature grain, with higher mycotoxin levels typically associated with nitrogen-poor treatments [23]. Additionally, the types of wheat cultivars can also influence the severity of FHB, with some showing higher resistance to the disease [24]. While fungicide treatments can reduce the frequency of FHB and mycotoxin concentrations in grain, their effectiveness depends on factors such as the temperature and timing of application. Effective management of FHB in wheat requires a comprehensive approach that considers factors such as the diversity of *Fusarium* species, weather conditions, the use of organic mulch, cultivar resistance, and proper fungicide application [25,26,27,28]. Central to implementing such strategies is the understanding of the specific *Fusarium* species present, their distribution, and the mycotoxins they produce. Therefore, the aim of this study was to investigate the prevalence, distribution, and species-specific mycotoxin production of *Fusarium* species associated with FHB in wheat fields across the Western Cape region of South Africa. Specifically, this study’s objectives are to (i) survey and identify *Fusarium* pathogens associated with wheat head blight in the West and South Coast districts of the Western Cape, (ii) provide an epidemiological report on FHB pathogens in these districts using disease severity and rating scales, (iii) isolate and characterize FHB pathogens through rDNA and species-specific PCR sequencing, and (iv) identify the mycotoxins produced by these FHB pathogens.

## 2. Materials and Methods

### 2.1. Field Survey and Sampling of Wheat for FHB Assessment in the Western Cape

**Figure 1:**
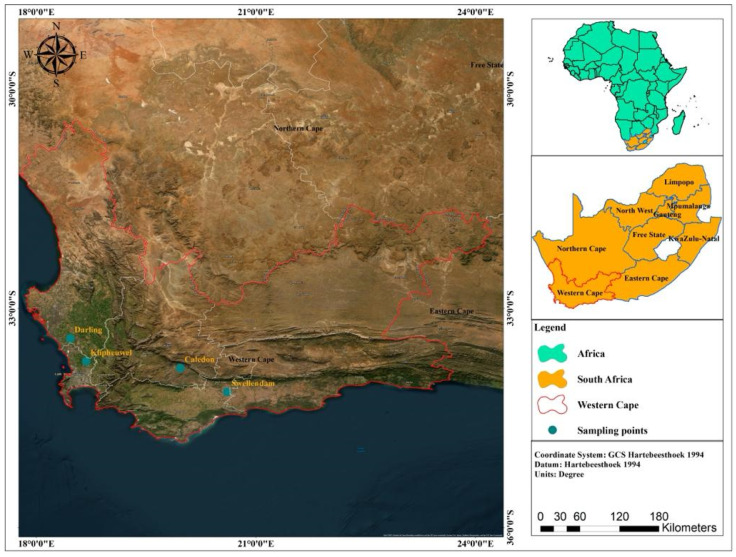
Map showing four sampling locations in the Western Cape region of South Africa.

A field survey of FHB disease was conducted between September and October 2022, and two fields each were selected in the West and South coast districts of the Western Cape. Darling and Klipheuwel represented the fields in the West coast, while Swellendam and Caledon represented the fields along the South coast (Figure 1). Each location was visited during the anthesis, milk, and dough development stages for sample collection. The pattern of sample collection involves a “W” pattern walk across the field, and 10 wheat heads from 10 sample points were randomly collected into a separate polythene bag for assessment in the laboratory.

Disease severity from the field was calculated using the relationship below:

$$Disease\;severity=\frac{Number\;of\;bleached\;head\;spikelets}{Total\;number\;of\;spikelets}\times 100$$

**Figure 2:**
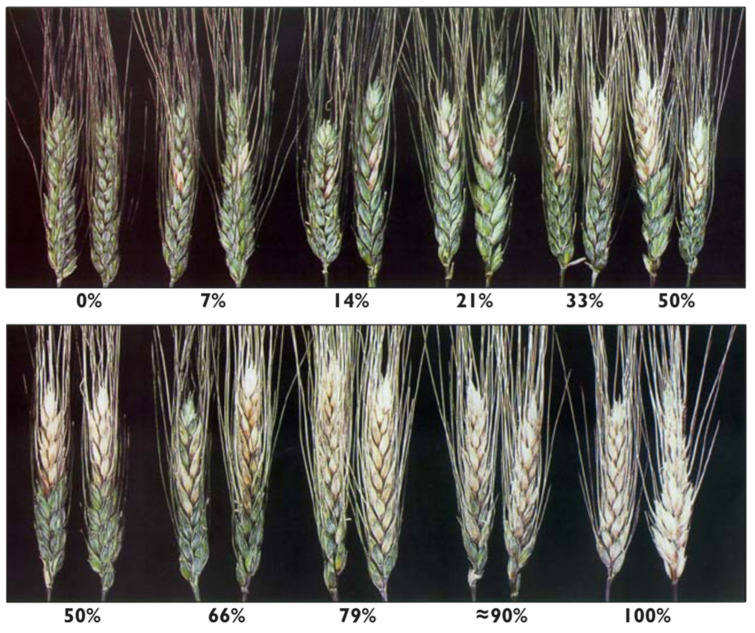
Fusarium head blight (FHB) severity rating scale in wheat showing percentage severity levels [29].

Wheat plants infected with head blight were identified based on visible symptoms on the spikelets, such as discoloration (turning pale or a bleached color, instead of the healthy green or yellow) and kernels that appeared shriveled, smaller, and lighter in weight. Wheat heads exhibiting blight symptoms were subsequently screened for FHB. All wheat spikes from each sampling location were counted and visually inspected for the presence or absence of FHB symptoms. Spikes were recorded as diseased when at least a single spikelet showed any signs of FHB, following the severity scale of Stack and McMullen [29] shown below (Figure 2).

The disease rate was calculated from the following relationship:

$$Disease\;rate=\frac{\begin{array}{c} Number\;of\;plants\;from\;category\;\left(1\right)\times their\;pathological\;evidence+\cdots \\ +\;number\;of\;plants\;from\;category\;(11)\times their\;pathological\;evidence \end{array}}{Total\;number\;of\;plants\;the\;highest\;pathological\;index}$$

To compare the development of wheat FHB over time, a disease regression equation for head blight severity across two months was derived using Microsoft Excel.

### 2.2. Isolation and Cultivation of Fusarium Isolates from FHB-Infected Wheat Spikelets

Following the identification of spikelets with FHB symptoms from each sample collection point, twenty kernels per spikelet with symptoms such as white or pinkish mold growth, bleached spikes, and scabby or shriveled kernels, were surface-sterilized with a 3% sodium hypochlorite solution for two minutes. The kernels were rinsed with sterile distilled water, placed in sterile tissue paper, and allowed to dry in the laminar flow cabinet. The surface-sanitized kernels were plated onto potato dextrose agar (PDA) amended with 200 mg·L^−1^ ampicillin and incubated for seven days at 25–28 °C to monitor the growth of any *Fusarium* species. *Fusarium* colonies were purified by selecting single spores and sub-cultured on fresh PDA plates to grow mycelium for DNA extraction.

### 2.3. DNA Extraction from Fusarium Isolates for Molecular Analysis

High-molecular-weight genomic DNA from the mycelia of single-spore *Fusarium* isolates was extracted using a Zymo Research Fungal/Bacterial DNA Mini Prep Kit (Epigenetics, Hatfield, South Africa) according to the manufacturer’s instructions. The DNA purity and concentration of each isolate were measured using a NanoDrop 2000^TM^ spectrophotometer (Thermo Scientific, Wilmington, NC, USA).

### 2.4. Polymerase Chain Reaction (PCR) Amplification of Fusarium Isolates

The ITS region of each *Fusarium* isolate (Table 1) was amplified using ITS1 (5′-TCCGTAGGTGAACCTGCG-3′) and ITS4 (5′-TCCTCCGCTTATTGATATGC-3′) primers [26]. The PCR reactions (25 µL) consist of 1 µL of 1:10 or 1:100 diluted DNA, 1 U RedTaq DNA polymerase (Sigma-Aldrich, Milan, Italy), 2 µL RedTaq buffer, 1.7 µL of 22 mM MgCl_2_ (final concentration: 3.0 mM), 10 mM dNTPs, and 1.0 µM of each primer (forward and reverse). The PCR amplifications conditions consist of an initial denaturation at 94 °C for 4 min, followed by 35 cycles of 94 °C for 60 s, 57 °C for 60 s, and 72 °C for 60 s, with a final extension at 72 °C for 5 min.

Using multiplex PCR with known species-specific primers (Table 1), *Fusarium* isolates were amplified using the reaction and amplification conditions previously described [30,31,32,33]. In addition, the genomic DNA of *F. ipomoeae* was amplified with species-specific primers (Table 1) using the reaction conditions previously described by Wang et al. [34] with slight modifications. The amplification conditions consist of an initial denaturation step at 95 °C for 2 min, followed by 35 cycles of denaturation at 95 °C for 45 s, annealing at 55 °C for 30 s, and extension at 72 °C for 1 min, with a final extension step of 72 °C for 7 min.

All PCR amplicons were electrophoresed on a 1.5% (*w*/*v*) agarose gel with a 1 kb DNA ladder (Bioline, Memphis, TN, USA) and visualized under UV light using ethidium bromide staining.

**Table 1:** Primer names and sequences of *Fusarium* species associated with wheat head blight.

Target	Primer	Primer Sequence (5-3)	T_a_ (°C)	Reference
*F. pseudograminearum*	Fp1-1	CGGGGTAGTTTCACATTTCCG	60	[30]
	Fp1-2	GAGAATGTGATGACGACAATA		
*F*. *culmorum*	Fc01F	ATGGTGAACTCGTCG TGG C	60	[31]
	Fc01R	CCC TTC TTA CGC CAA TCT CG		
*F. graminearum*	Fg16F	CTC CGG ATA TGT TGC GTC AA	60	[31]
	Fg16R	GGT AGG TAT CCG ACA TGG CAA		
*F. avenaceum*	FaF	CAA GCA TTG TCG CCA CTC TC	60	[35]
	FaR	GTT TGG CTC TAC CGG GAC TG		
*F*. *equiseti*	FeF1	CATACCTATACGTTGCCTCG	60	[32]
	FeR1	TTACCAGTAACGAGGTGTATG		
*F. tricinctum*	FtricACL1-f	CTG TGT GTT TGG TGG GAT TGG	60	[33]
	FtricACL1-r	TGG GAG TAG ACC GGG AAA AC		
*F. ipomoeae*	CL1F	GARTWCAAGGAGGCCTTCTC	55	[34]
	CL2R	TTTTTGCATCATGAGTTGGAC		

Ta = Annealing temperature.

### 2.5. PCR Amplicon Sequencing and Phylogenetic Analysis

PCR products (ITS and species-specific) for each *Fusarium* isolate were purified and sequenced at Central Analytical Facilities of Stellenbosch University (Stellenbosch, South Africa). To identify the *Fusarium* isolates, the DNA sequences were edited and aligned using the Cluster W multiple sequence alignment tool in Molecular Evolutionary Genetics Analysis (MEGA) 11.0.13 software [36] and compared to sequences in the NCBI GenBank database (http://blast.ncbi.nlm.nih.gov, accessed on 12 September 2024). A phylogenetic tree was constructed using 500 bootstrap replicates and the maximum likelihood technique. Based on scores for the Bayesian information criterion, the molecular evolution model that best suited the data was chosen [37].

### 2.6. Profiling of Mycotoxin Chemotypes in the Fusarium Species

To profile for nivalenol (NIV) and 15-acetyl-deoxynivalenol (15-ADON) chemotypes, the protocol from van Coller et al. [1] was adopted, while for the zearalenone (ZEN) chemotype, the protocol from Neera and Murali [38] was adopted. *Fusarium* species chemotypes were profiled using multiplex PCR assays to amplify portions of the respective genes for the different mycotoxins.

## 3. Results

### 3.1. Identification of Wheat Head Blight Pathogens in Western Cape Region of South Africa

**Figure 3:**
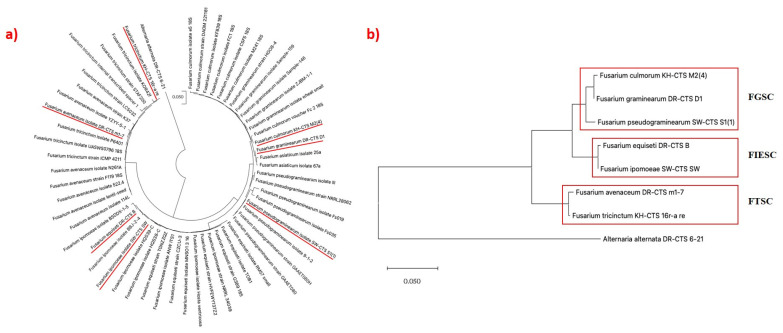
(**a**) Maximum likelihood phylogenetic analysis of ITS-rDNA sequences showing the relationship between *Fusarium* isolates with reference sequences from NCBI. The phylogenetic tree shows the relationships of *Fusarium* isolates causing FHB disease in wheat. The identified *Fusarium* isolates used in this study are underlined in red. (**b**) Phylogenetic tree showing relationship between *Fusarium* isolates in different species complexes (FGSC, FIESC, FTSC) associated with FHB. The sequence of *Alternaria alternata* DR-CTS 6-21 was used as an outgroup. Keys: FGSC = *Fusarium graminearum* species complex; FIESC = *Fusarium incarnatum-equiseti* species complex; and FTSC = *Fusarium tricinctum* species complex.

To identify the *Fusarium* isolates used in this study (Table 2), genetic polymorphism in the DNA sequences were analyzed. The DNA sequences of each *Fusarium* isolate have been deposited into GenBank and under different accession numbers (Table 2). Phylogenetic analysis revealed that *Fusarium culmorum* and *Fusarium graminearum* form a clade with *F. culmorum* voucher Fc 2 18s, whereas *F. equiseti* and *F. ipomoeae* were closely clustered and formed another clade with *F. equiseti* isolates RT-B2. The remaining three strains, *F. pseudograminearum*, *F. avenaceum*, and *F. tricinctum* formed monophyletic clades (Figure 3a).

The seven *Fusarium* isolates were grouped into three different complexes (Figure 3b). The first complex, *Fusarium graminearum* species complex (FGSC), consists of *F. culmorum*, *F. graminearum*, and *F. pseudograminearum.* The second complex, *Fusarium incarnatum-equiseti* species complex (FIESC), consists of *F. equiseti* and *F. ipomoeae*, while the *Fusarium tricinctum* species complex (FTSC) consists of *F. avenaceum* and *F. tricinctum.*

**Figure 4:**
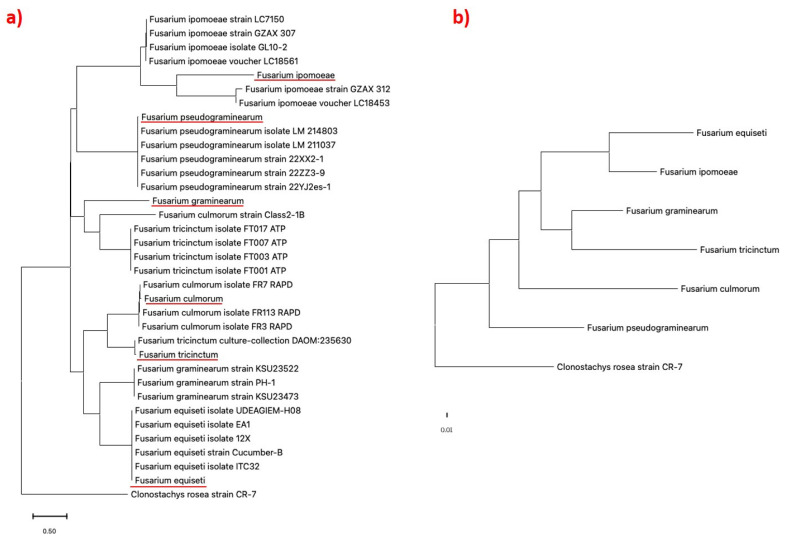
(**a**) Maximum likelihood phylogenetic analysis of species-specific gene sequences showing the relationship between *Fusarium* isolates with reference sequences from NCBI. The tree shows the phylogenetic relationships of *Fusarium* species causing FHB disease in wheat. The identified *Fusarium* isolates used in this study are underlined in red. (**b**) Comparative phylogenetic analysis of *Fusarium* isolates using species-specific gene sequences. The sequence of *Clonostacys rosea* strain CR-7 was used as an outgroup.

Using species-specific primers, the phylogenetic analysis of the different *Fusarium* isolates shows clustering with the different *Fusarium* species from the NCBI database (Figure 4a).

Phylogenetic analysis revealed close relationships among the isolates: *F. ipomoeae* with strain GZAX 312, *F. pseudograminearum* with strain LM 214803, *F. culmorum* with strain FR113 RAPD, *F. tricinctum* with strain DAOM:235630, and *F. equiseti* with strain ITC32 (Figure 4a). A comparative phylogenetic analysis between the different *Fusarium* species (Figure 4b) shows that the isolates differ from one another with the closely related species originating from the same root in the tree.

### 3.2. Disease Severity and Rate from the Sampling Locations in the Western Cape Region

**Figure 5:**
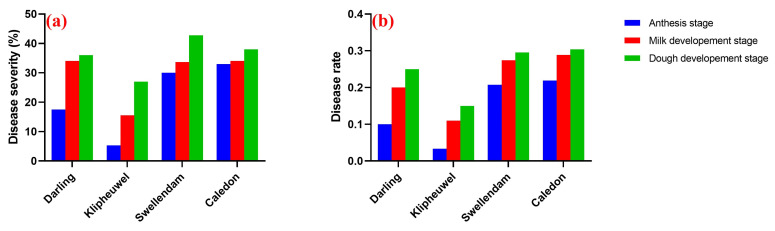
FHB disease severity (**a**) and disease rate (**b**) across different sampling locations.

**Figure 6:**
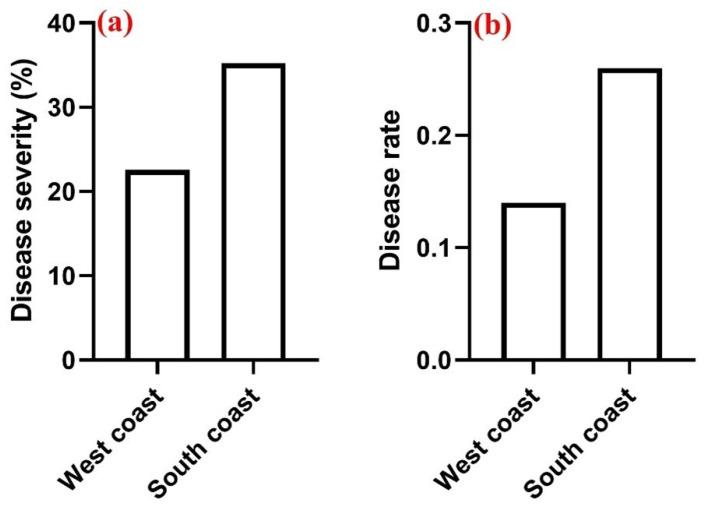
FHB disease severity (**a**) and disease rate (**b**) across the two sampling regions.

Swellendam in the South coast recorded the highest disease severity (42.73%), followed by Caledon (38.00%), while Klipheuwel and Darling in the West coast had disease severities of 27.00% and 36.00%, respectively, at the dough development stage (Figure 5a). The disease progression at different stages of wheat development was also measured across the four sampling locations, and the results indicate that the dough stage showed the highest disease rate in all locations (Figure 5b). Swellendam and Caledon in the South Coast region showed the highest disease rate (0.3), followed by Darling (0.25) and Klipheuwel (0.15) in the West Coast region (Figure 5b). In addition, both disease severity and rate were higher in the South Coast of the Western Cape (35.22% and 0.26%, respectively) compared to the West Coast (25.56% and 0.14%, respectively) (Figure 6a,b).

### 3.3. Frequency and Distribution of Different Fusarium Species Across the Different Sampling Locations

**Figure 7:**
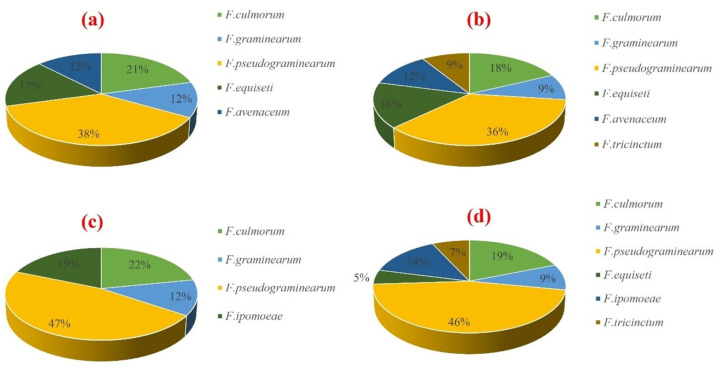
Frequencies of all *Fusarium* species identified from (**a**) Darling, (**b**) Klipheuwel, (**c**) Swellendam, and (**d**) Calendon.

The abundance of individual FHB-related species varied across the different sampling locations (Figure 7a–d), with fewer isolates identified in Swellendam (Figure 7c). *F. ipomoeae* was identified exclusively in Swellendam and Caledon, in the South coast region, with frequencies of occurrence of 19% and 14%, respectively (Figure 7c,d). On the other hand, *F. tricintum* was only identified in Klipheuwel and Caledon, with occurrence frequencies of 36% and 46%, respectively (Figure 7b,d). The remaining species including *F. culmorum*, *F. graminearum*, *F*, *pseudograminearum*, *F. equiseti*, and *F. avenaceum* were present in all sampling locations (Figure 7a–d).

### 3.4. Profiling of Mycotoxin Chemotypes by Fusarium Species Isolated from FHB Diseased Wheat Spikelet

A total of 112 *Fusarium* isolates were identified from the spikelets of diseased wheat plants (n = 1200) and characterized into seven different *Fusarium* species across the four sampling locations (Table 3). The seven *Fusarium* species were further characterized to identify their mycotoxin chemotypes. *F. culmorum* produced zearalenone (ZEN) and nivalenol (NIV) chemotypes, *F. pseudograminearum* produced ZEN and 15-acetyl-deoxynivalenol (15-ADON), while *F. tricinctum* only produced NIV (Table 3). None of the tested mycotoxins were detected in *F. graminearum*, *F. equiseti*, *F. ipomoea*, and *F. avenaceum.*

## 4. Discussion

Seven *Fusarium* species from seven of the major *Fusarium* species complexes were associated with FHB of wheat from all the sampling locations in the Western Cape region of South Africa (Table 2, Figure 3a,b). Most of the isolates consist of species from FGSC. Furthermore, phylogenetic analysis of the different isolates using specific gene primers shows a perfect clustering of the individual isolates with their corresponding species from the database (Figure 4a). Comparative phylogenetic analysis between the respective isolates shows that they all differ from one another with the closely related species originating from the same branch in the phylogenetic tree (Figure 4b). This analysis was necessary to ensure that the individual species of *Fusarium* completely differs from one another. The findings from this study agree with the reports of Van Coller et al. [1] and Minnaar-Ontong et al. [14], demonstrating FGSC as major FHB pathogens of wheat in South Africa and one of the major pathogens associated with head blight in the world [14,21,39,40,41,42,43,44]. Over three decade ago, the outbreak of FHB disease associated with FGSC was recorded in South Africa in KwaZulu-Natal and parts of Bushveld in the Eastern region of the Free State [45]. Ten years later, different batches of FHB-infected wheat seeds collected from fields at Prieska (Northern Cape) reported the presence of *F. equiseti* and *F. culmorum* as part of the identified pathogens from the seeds [14]. *Fusarium avenaceum* and *F. pseudograminearum* identified from in this study were among the first reported FHB pathogens in wheat identified from South Africa in 2022 [1].

The outbreak of FHB disease depends largely on the environmental and weather conditions which can vary between seasons. The disease severity was measured in each field during the different stages of wheat development, and the results show an increase in the severity and rate of the disease from the pollination to dough stage across the four different fields (Figure 5a,b and Figure 6a,b). Increase in the severity and rate of the disease could be attributed to different climatic factors such as temperature and rain, which favor the outbreak of FHB particularly in wheat [46]. Furthermore, increase in the severity of the disease across the different developmental stages could be due to the type of farming practice (types of crops planted on the field in the previous years). For example, a large amount of within-field maize residue is an FHB risk factor. Therefore, rotational practice involving no-host crops is recommended for FHB management [47,48]. Though we did not quantify the surface residue of FHB in this study, it was also reported that no-till systems typically leave about 30% of the soil surface covered with residue [46]. Besides the farming practice method, the severity of the disease could have been influenced by cultivar resistance and management practices such as fungicide application [49,50]. A major challenge in the management of FHB disease in South Africa is the unavailability of resistant wheat varieties, bio-fungicides, and licensed fungicides to the local farmers [3].

Studies have shown that there is a strong correlation between favorable climatic conditions such as temperature and high humidity with FHB epidemics before and during flowering stage [51,52]. At the time of this survey between September and October 2022, the average temperature was between 13 °C (55 °F) and 21 °C (70 °F), with a high humidity of 73% in Cape Town, which may favor the growth of *Fusarium* species (Figure 7). Another factor that may favor a high occurrence of these pathogens is the stage of the plants (dough stage) when the samples were collected, as FHB symptoms are most pronounced, but kernels are not fully developed. During isolations, it may be difficult to determine which kernels are diseased when they are dry a few days after sampling [1]. An interesting observation from this study is the presence of *F. culmorum*, *F. graminearum*, *F. pseudograminearum*, *F. equiseti,* and *F. avenaceum* from all the sampled locations (Figure 7). A recent study conducted in the Western Cape region showed that over 80% of the isolates associated with FHB disease symptoms were identified as *F. pseudograminearum*, the major pathogen in the study. However, contrary to their report, *F. pseudograminearum* isolated in this study produces zearalenone and 15-ADON chemotypes, but not the NIV chemotype [1,43,53,54], while *F. culmorum* produces both the NIV and ZEN chemotypes (Table 3). Surveillance studies should include the determination of *Fusarium* mycotoxins and their acetylated derivatives, as this can offer valuable information on the distribution of toxigenic strains of the fungus, which may pose significant threats to food safety and security.

## 5. Conclusions

This study isolated and identified seven distinct strains of *Fusarium* from wheat samples collected across various wheat-growing regions to assess the prevalence and severity of FHB. Phylogenetic analysis grouped these strains into three major complexes: FGSC, FIESC, and FTSC, demonstrating clear geographical variation in species distribution. The results revealed that FHB development peaked during the dough stage of wheat growth, with the highest disease incidence recorded in Swellendam and Caledon in the southern region of South Africa. Mycotoxin profiling highlighted the production of key toxins, including ZEN, 15-ADON, and NIV by the isolated *Fusarium* strains, underscoring the significant threat FHB poses to food safety. These mycotoxins are known to contaminate wheat products, posing health risks to consumers and potentially affecting trade and market access. The detection of *F. ipomoeae* and *F. tricinctum* in specific locations, along with the high frequency of FGSC members across all sampled areas, underscores the need for targeted and region-specific management strategies. It is worth noting that the detection of *F. Ipomoeae* in the spikelets of diseased wheat heads represents the first report globally, while the identification of *F. tricinctum* is the first report in South Africa. The identification of toxigenic strains capable of producing multiple mycotoxins highlights the importance of continuous monitoring to alleviate the risks posed by FHB to both food safety and food security. Further research should focus on the development and use of resistant wheat cultivars, optimizing fungicide application timing, and exploring novel biocontrol measures to manage FHB effectively. Long-term epidemiological studies under varying climatic conditions are also important to better understand disease dynamics and improve control strategies. This study provides a crucial foundation for policy development aimed at safeguarding wheat production, improving food safety, and ensuring sustainable agricultural practices in South Africa.

## Figures and Tables

**Table 2:** GenBank accession numbers for *Fusarium* species used in phylogenetic analysis.

*Fusarium* Species	GenBank Accession
*Fusarium avenaceum* DR-CTS M1-7	PQ192151
*Fusarium culmorum* KH-CTS M2(4)	OR580986
*Fusarium equiseti* DR-CTS B	OR581616
*Fusarium graminearum* DR-CTS D1	OR582334
*Fusarium ipomoeae* SW-CTS SW	OR582482
*Fusarium pseudograminearum* SW-CTS S1(1)	OR582866
*Fusarium tricinctum* KH-CTS 16r-a re	PQ192641

**Table 3:** Profiling mycotoxin chemotypes of *Fusarium* species isolated from FHB diseased wheat spikelet.

Fusarium Species	ZEN	15-ADON	NIV
*F. culmorum* KH-CTS M2(4)	+	-	+
*F. graminearum* DR-CTS D1	-	-	-
*F. pseudograminearum* SW-CTS S1(1)	+	+	-
*F. equiseti* DR-CTS B	-	-	-
*F. ipomoeae* SW-CTS SW	-	-	-
*F. tricinctum* KH-CTS 16r-a re	-	-	+
*F. avenaceum* DR-CTS M1-7	-	-	-

## Data Availability

Data are contained within the article.
